# Partnering With Schools for Community-Based Health Interventions: How Educating Children Improves Hypertension Awareness

**DOI:** 10.31486/toj.24.0099

**Published:** 2025

**Authors:** Jennifer Hundley, Kristine Olson, Cherylann Rocha, Margaret K. Wallace, Grace Smith, Katharina Martin, Micheal Crane, Ralph D’Agostino, Amy Ladd, Sangeeta Shah

**Affiliations:** ^1^ Division of Cardiology, Department of Internal Medicine, VCU Health, Richmond, VA; ^2^ VCU School of Education, Richmond, VA; ^3^ Virginia Commonwealth University, Richmond, VA; ^4^ The College of William and Mary, Williamsburg, VA; ^5^ Lower School, Anna Julia Cooper School, Richmond, VA; ^6^ Biostatistics and Data Science, Wake Forest University, Winston-Salem, NC

**Keywords:** *Adolescent*, *health education*, *health educators*, *hypertension*, *public health*, *schools*, *students*

## Abstract

**Background:**

More than 16,000 Virginians die of cardiovascular disease each year, with increased morbidity among Black and low-income adults. Hypertension (HTN) is the most modifiable cardiovascular disease risk factor. A community-based health intervention administered in partnership with schools may increase HTN awareness and reduce the development of unhealthy practices.

**Methods:**

Elementary school students (n=52) attending a majority Black and low-income school participated in an educational intervention program called Teach BP that is designed to increase HTN awareness across 4 topics: knowledge of blood pressure (BP) and HTN, organ systems impacted by HTN, habits to maintain a healthy BP, and competency in operating a BP monitor.

**Results:**

Students’ ability to define and recognize HTN increased by an average of 62.7%. Their awareness of how HTN affects the body increased by an average of 92.1%. Additionally, students demonstrated competency in operating a BP monitor.

**Conclusion:**

The Teach BP program was effective at increasing students’ awareness of HTN.

## INTRODUCTION

Hypertension (HTN) is the term used for a blood pressure (BP) reading higher than 130/80 mm Hg. If left untreated, HTN can cause organ damage and lead to heart complications, kidney failure, vision loss, and strokes.^1^ More than 16,000 Virginians die of cardiovascular disease each year.^2^ HTN disproportionately affects certain communities. In 2020, in Richmond, Virginia, 341 deaths of adults aged 35+ years occurred from heart disease per 100,000 individuals. This number increases to 477 deaths among the Black population.^3^ Across the United States during the period 2017 to 2020, 58.4% of Black non-Hispanic females and 57.5% of Black non-Hispanic males aged 20+ years had HTN compared to 42.6% of non-Hispanic White females and 48.9% of non-Hispanic White males.^4^ Children with parents and guardians who have HTN are more likely to develop HTN themselves.^5^ In addition, sedentary lifestyles, obesity, and poverty are associated with an increased risk of HTN.^6,7^ Among Richmond, Virginia, adults, 31.9% are obese, 27% are considered physically inactive, and 18.8% live in poverty (with an income below 150% of the federal poverty level), all of which exacerbate the risk for HTN.^8,9^

Awareness is an important consideration, especially because HTN in early stages does not present with symptoms. In the United States during the period 2017 to 2020, 62.0% of individuals were aware of their diagnosis, 52.6% were receiving treatment, and only 25.7% had obtained HTN control.^4^ Young people are the least likely to be aware of their diagnosis, with the lowest awareness among 20- to 39-year-olds (37.6%).^4^

Effective strategies for decreasing HTN morbidity and mortality are to raise community awareness and to deliver proper treatment for control. Interventions targeting these strategies can potentially improve the health outcomes of individuals with HTN.

### Engaging a Community to Promote Public Health

Community-based health interventions are an effective model for increasing awareness, treatment, and control of HTN in at-risk individuals. The onset of HTN affects a diverse range of individuals, and because HTN is influenced by the living environment,^10^ growing evidence indicates that community-based approaches can provide effective interventions tailored to the experiences of a specific population.^11-13^ Community-based health interventions rely on the interaction between community health workers and the population of interest. This interaction is often established by implementing programs directly in community spaces. A review of community-based health interventions in urban neighborhoods found that 75.0% of programs reported improvements in at least one desired parameter.^14^ Changes are most significant among small studies that target populations with pervasive health issues.^15-20^

### Empowering Students With Health Interventions

Schools are essential community spaces that connect administrators, teachers, students in kindergarten through twelfth grade (K-12), and their families on a consistent and daily basis. K-12 schools are an ideal setting for community-based health interventions because they provide a unique opportunity for early learning and intervention. International research has shown that schools play a vital role in facilitating behavior change to prevent noncommunicable diseases.^21^ In a survey of school-based health interventions, Sliwa et al concluded that such interventions are feasible in a variety of settings, including racially diverse and low-income schools.^22^ Further, school-based health interventions have successfully transferred declarative knowledge about public health to children.^23^ By targeting adolescents, a school-based health intervention has the potential to prevent the onset of chronic diseases and promote lifelong wellness by motivating children to establish healthy habits and lifestyle choices.^22^

While research that examines the efficacy of HTN interventions with students in the United States is limited, researchers in other countries have successfully used school settings to further awareness. In Bogota, Colombia, Céspedes et al engaged preschool students (n=1,216) in a curriculum designed to increase knowledge about cardiovascular health.^24^ Preschoolers exposed to the curriculum generated a 10.9% increase in survey scores that measured knowledge, attitudes, and habits.^24^ Similarly, a 2020 study based in South India evaluated the efficacy of an HTN education module on increasing awareness, control, and treatment for children (n=110) and their parents (n=100).^25^ Both groups improved significantly on post-program assessments across a variety of criteria, such as understanding HTN risk factors, symptoms, and complications.^25^ A program conducted in Singaporean elementary schools engaged fifth-grade students (n=3,926) in learning about normal and elevated BP levels, HTN risk factors, prevention of HTN, and how to take an automated BP.^26^ Students were instructed to share what they learned with their parents, and each student shared with an average of 3.04 family members, effectively tripling the distribution of HTN knowledge.^26^

Despite high rates of HTN in the Richmond, Virginia, region, the Richmond Public Schools wellness policies lack any mention of HTN awareness.^27,28^ This disparity, in addition to the success of interventions reported in the literature, identified Richmond Public Schools as a promising site for a community-based health intervention.

### Study Aim

This study aimed to determine the feasibility of implementing an HTN-focused community-based health intervention in an elementary school. We wanted to determine if the community-based health intervention—Teach BP—administered during class time could successfully increase HTN awareness among elementary school students.

## METHODS

This project was approved by the Virginia Commonwealth University Institutional Review Board with exempt status as the Teach BP curriculum was approved by the school and did not alter the students’ school requirements.

### Study Site and Participants

The study was conducted at an independent school in East Richmond, Virginia, that serves low-income families and provides full tuition scholarships to students in kindergarten through eighth grade.^29^ The school was selected because it serves low-income students, and the student body is 96.4% African American.^30^ These demographics are characteristic of a population disproportionately impacted by HTN. The study involved 2 cohorts of students from fourth to sixth grade.

### Procedure

The study was composed of 2 phases. During phase I in spring 2022, we taught a group of fourth and fifth graders, termed the original cohort. During phase II in fall 2022, we taught the original cohort as fifth and sixth graders and a new fourth-grade class called the subsequent cohort.

Classroom time was used to implement an experiential learning curriculum called Teach BP. Developed by the VCU Health Pauley Heart Center, Richmond, Virginia, Teach BP is designed to increase HTN awareness across 4 topics: knowledge of BP and HTN, organ systems impacted by HTN, habits to maintain a healthy BP, and competency in operating a BP monitor. The study team included a lead instructor, volunteer field teachers (1 for up to 5 students), and a program coordinator who was responsible for arranging session logistics. Study team members were Pauley Heart Center and Virginia Commonwealth University staff or students.

**Figure 1.**
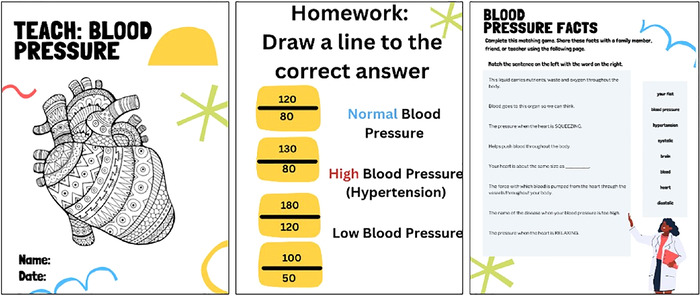
Each student received a Teach BP workbook. The workbook cover is shown on the left, and the 2 pages show complementary activities designed to reinforce knowledge of blood pressure definitions. The workbooks were created using Canva Pro (Canva).

Over the course of a week, the Teach BP team conducted 4 classes, each an hour long. Program instrumentation included an introduction video, didactic slides, a Teach BP workbook (Figure 1), interactive activities, and a group project related to heart health called the Changemaker project. Activities were designed to teach BP terminology, the components of a balanced lifestyle, and the dangers of HTN. The lead instructor taught students how to take a BP reading, and students practiced on their field teachers and peers. The initial program was identical for both cohorts, but the original cohort received a 1-hour refresher in phase II (fall 2022).

### Assessment Time Points

**Figure 2.**
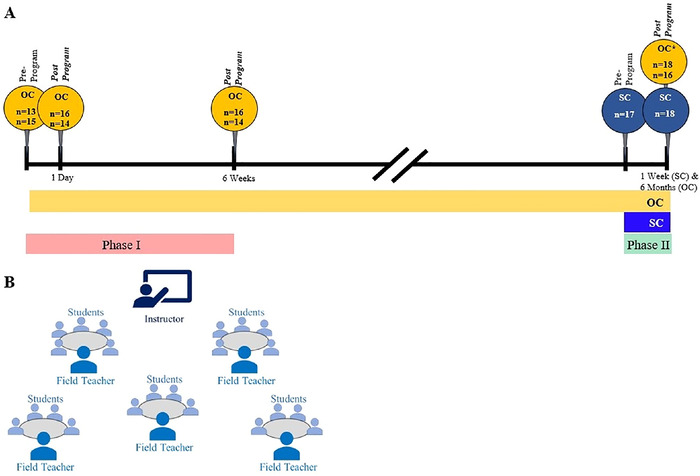
(A) Two student cohorts—the original cohort (OC) and the subsequent cohort (SC)—participated in phases I and II of the Teach BP intervention. The yellow and blue rectangles under the timeline show the total length of time the 2 cohorts were followed, the pink rectangle shows the time allotted to phase I, and the green rectangle shows the time allotted to phase II. The OC completed phase I as fourth and fifth graders and completed phase II as fifth and sixth graders; the yellow circles identify the survey assessment points (pre-program and post-program) for the OC and the sample sizes at each assessment point. The OC received a refresher course during phase II, indicated by the asterisk in the yellow circle. The SC was a new fourth-grade class that completed phase II only; the blue circles identify the survey assessment points (pre-program and post-program) for the SC and the sample sizes at each assessment point. (B) In the Teach BP classroom, an instructor provided oversight and taught the lesson to the whole group, while field teachers worked with groups of up to 5 students.

To measure their comprehension of the curriculum, students completed pre-program and post-program surveys at multiple assessment time points. The original cohort completed surveys pre-program, 1 day post-program, 6 weeks post-program (in spring 2022), and 6 months post-program (in fall 2022). The subsequent cohort completed surveys before receiving any instruction and 1 week post-program. Student attendance varied on the days the Teach BP team performed time point assessments. Figure 2 shows the study timeline, student sample sizes at each assessment time point, and the classroom configuration.

The Teach BP team also administered rubric checks to measure students’ procedural knowledge for how to use a BP monitor. During the rubric checks, students were asked to take a field teacher's BP using a BP monitor and to interpret the results. The original cohort had rubric checks at 6 weeks and 6 months post-program. The subsequent cohort had their rubric check at 1 week post-program.

### Survey Instrumentation

The surveys assessed comprehension of 3 broad topic areas: (a) “How can diet & exercise affect HTN?”, (b) “What is BP & HTN?”, and (c) “How can HTN affect your body?”. A correct response to a question resulted in a +1 score, while an incorrect answer, a don’t know answer, or an unanswered question resulted in a 0 value.

Following the comprehension questions, students ranked statements intended to measure their self-efficacy and attitudes regarding taking a BP measurement.

The students rated 9 statements to assess self-efficacy related to taking a BP. Responses ranged from 1 (strongly disagree) to 5 (strongly agree). Self-efficacy statements were designed to reflect students’ beliefs in their own capability to complete a specific action related to BP.^31^ The self-efficacy section included statements such as “I am confident in my ability to measure my own BP.”

Students also ranked 4 statements regarding their attitudes on taking a BP measurement. Attitude statements measured students’ beliefs about the importance of taking BP to assess a person's health. Students rated attitude statements such as “For me, taking BP measurement is…” on a scale of 1 to 5, with the numeric values representing different concepts for each statement (eg, “useless” to “useful” or other contrasting terms).

To evaluate the results of the self-efficacy and attitude statements, student responses of 4 or 5 were scored as +1, while responses of 1, 2, or 3 were scored as 0. With 9 self-efficacy statements, the maximum possible score was 9. With 4 attitude statements, the maximum possible score was 4.

The surveys were modified for clarity before being administered during phase II. Changes in wording and format were made to better suit the grade level understanding of the students. For example, multipart questions were broken into individual parts, and the order of questions was reorganized.

Surveys were administered in person as paper quizzes. A field teacher read each question aloud and asked for verbal confirmation that each student had completed the survey before collecting them.

To evaluate their ability to accurately take a BP reading, students were scored on an 8-step rubric that assigned a score of 1 for a correctly done task and a score of 0 for an incomplete or incorrect action. No rubric measurements were performed pre-program, as giving the students a BP cuff (a medical device) without adequate instruction may have resulted in inaccurate and potentially harmful use of the device.

### Data Analysis

Survey assessment data were collected from the original cohort during phase I of the program. During phase II, data at the 6-month time point were collected but were not incorporated with the statistical analyses of phase I. For continuous outcomes (ie, the sum of the 11 pre-program “What is BP & HTN?” questions) of phase I original cohort data, we fit a series of 2-way analysis of variance (ANOVA) models to examine each outcome separately. In these models, we included 2 fixed effects—time (pre-program, 1 day post-program, or 6 weeks post-program) and grade (fourth or fifth)—and the time by grade interaction. If the interactions were not significant (*P*>0.15), we refit each model with the main effects of time and grade.

Because individual participants were not tracked in the original cohort—classes were tracked instead—these models did not examine the effects of the intervention on students through time but examined the overall changes over time, adjusting for the grade level of the students.

Survey assessment data from the subsequent cohort were collected during phase II at 2 time points: pre-program and 1 week post-program. The pre-program and 1-week post-program survey results for the subsequent cohort were tracked with identifiers, and the data were compared using paired *t* tests.

Competency in taking a BP measurement was not statistically analyzed but is displayed as the percentage of students who correctly demonstrated each of the competency skills.

Analyses and graphical representations of the data were performed using Prism version 10 (GraphPad Software Inc). All analyses were performed using 2-sided tests, and *P* values <0.05 for main effects and <0.15 for interactions were considered statistically significant.

## RESULTS

### Participation

During phase I, the original cohort students generated 28, 30, and 30 survey responses at the pre-program, 1-day post-program, and 6-week post-program time points, respectively (Figure 2). During phase II, we collected 34 original cohort student responses at the 6-month time point. Data from this time point were not included in the analyses because of survey revision.

During phase II, the subsequent cohort generated 17 and 18 survey responses at the pre-program and 1-week post-program time points, respectively (Figure 2).

### Original Cohort Outcomes

#### Survey Assessment Data.

At the 3 phase I time points, all 2-way ANOVA models found nonsignificant *P* values (ie, >0.15) for the grade by time interactions in the original cohort. The impact of the intervention over time was not statistically different by grade.

**Figure 3.**
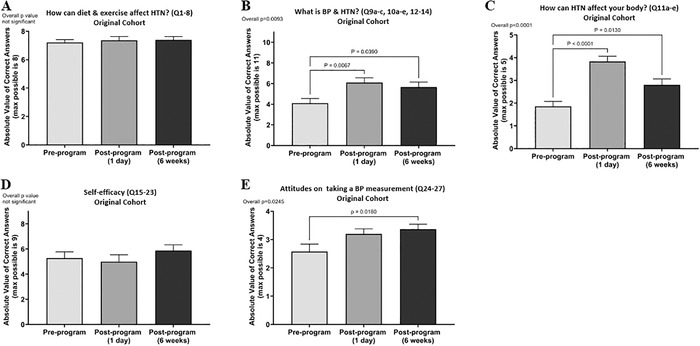
**Students in the original cohort were assessed at 3 time points on their knowledge of (A) “How can diet and exercise affect HTN?”, (B) “What is BP & HTN?”, and (C) “How can HTN affect your body?”. The students were also asked to rate statements showing (D) self-efficacy and (E) attitudes on taking a blood pressure measurement. Bars represent the mean ± standard error of the mean. One-way analysis of variance was used to detect differences among the time points. For assessments with a significant overall *P* value, a Dunnett multiple comparisons test was conducted with the pre-program assessment as the control time point compared to each post-program time point. Adjusted *P* values from the multiple comparisons are listed above the brackets.** BP, blood pressure; HTN, hypertension; max, maximum; Q, question.

The original cohort showed a high level of pre-program knowledge about the topic “How can diet & exercise affect HTN?” with an average score of >7 (of 8 questions), and no significant change was seen over time (Figure 3A). The topic “What is BP & HTN?” (11 questions) had an average score of 4.1 pre-program, which rose significantly to 6.1 at 1 day post-program (*P*=0.0067) and decreased to 5.7 at 6 weeks post-program (*P*=0.0390) (Figure 3B). The topic “How can HTN affect your body?” (5 questions) had an average score of 1.9 pre-program, which rose significantly to 3.8 at 1 day post-program (*P*<0.0001) and decreased to 2.8 at 6 weeks post-program (*P*=0.0130) (Figure 3C).

Self-efficacy was assessed with 9 statements. We found no significant differences between the time points, with average scores of 5.3 (pre-program), 5.0 (1 day post-program), and 5.9 (6 weeks post-program) (Figure 3D).

Attitudes on taking a BP measurement were assessed with 4 statements The average scores improved from 2.6 (pre-program) to 3.2 (1 day post-program) and then rose significantly to 3.4 (6 weeks post-program) (Figure 3E).

The original cohort completed a 6-month follow-up survey during phase II. As previously noted, the survey was revised to improve clarity based on feedback from phase I (eg, dividing a multipart question into separate questions). Because the phase II survey questions were slightly different, we did not add the results to Figure 3 and instead report the data as an independent time point. The 34 students who took the 6-month post-program survey demonstrated knowledge similar to their knowledge at the 1-day and 6-week post-program time points: “How can diet & exercise affect HTN?” (7.7 average on 8 questions); “What is BP & HTN?” (4.8 average on 8 questions); and “How can HTN affect your body?” (3.5 average on 5 questions). The self-efficacy average score of 6.5 on 9 statements was higher than the scores at the prior post-program time points, while the attitudes on taking a BP measurement average score of 2.8 on 4 statements closely mirrored the pre-program score.

#### BP Competency Rubric Data.

**Figure 4.**
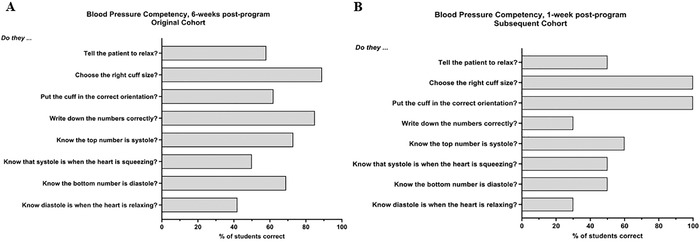
An 8-item rubric was used to assess students’ competency in taking a blood pressure measurement. (A) Students in the original cohort (n=26) were assessed at 6 weeks post-program. (B) Students in the subsequent cohort (n=10) were assessed at 1 week post-program.

A rubric completed at 6 weeks post-program during phase I (n=26) assessed the students’ ability to take a BP measurement. The rubric was conducted one-on-one with students, and because of staffing and time limitations, not all students were able to complete the rubric. Results are reported as the percentage of students who correctly demonstrated the step. Of the 8 rubric steps, the original cohort scored >55% on 6 steps: “Tell the patient to relax?” (58% [15 students]), “Choose the right cuff size?” (89% [23 students]), “Put the cuff in the correct orientation?” (62% [16 students]), “Write down the numbers correctly?” (85% [22 students]), “Know the top number is systole?” (73% [19 students]), and “Know the bottom number is diastole?” (69% [18 students]) (Figure 4A).

Thirty original cohort students were assessed for BP competency at the 6-month time point during Phase II and scored >55% on 6 of the 8 steps: “Tell the patient to relax?” (60% [18 students]), “Choose the right cuff size?” (90% [27 students]), “Know the top number is systole?” (73% [22 students]), “Know the bottom number is diastole?” (77% [23 students]), “Know that systole is when the heart is squeezing?” (57% [17 students]), and “Know diastole is when the heart is relaxing?” (63% [19 students]) (data not shown). Competency in 4 steps overlapped with the prior time point.

### Subsequent Cohort Outcomes

#### Survey Assessment Data.

**Figure 5.**
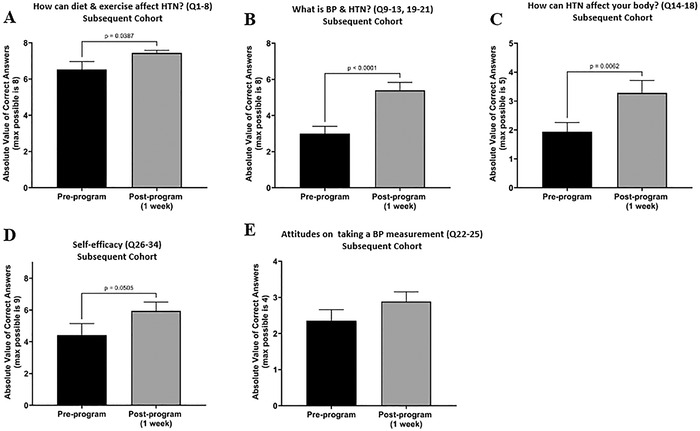
**Students in the subsequent cohort were assessed at 2 time points on their knowledge of (A) “How can diet and exercise affect HTN?”, (B) “What is BP & HTN?”, and (C) “How can HTN affect your body?”. The students were also asked to rate statements showing (D) self-efficacy and (E) attitudes on taking a blood pressure measurement. Bars represent the mean ± standard error of the mean. Completing the surveys were n=17 students pre-program and n=18 students post-program, with the 17 matching pairs analyzed by paired 2-tailed parametric *t* tests. Comparisons with significant *P* values are listed above the brackets in panels A, B, and C. Panel D shows a nonsignificant *P* value trending toward significance.** BP, blood pressure; HTN, hypertension; max, maximum; Q, question.

The subsequent cohort, a group of fourth graders, participated during phase II of the program. Their pre-program and 1-week post-program survey results were tracked with identifiers, so paired *t* tests were used to analyze the 17 matching pairs. The 1-week post-program time point included an additional student with no pre-program data (Figure 2). That student's data are included in the 1-week post-program bars in Figure 5, but the *P* values listed are based on paired *t* tests of the 17 pairs. In the results below, we report two 1-week average scores: one score excluding the 18th participant and one score including the participant.

We assessed the subsequent cohort on their knowledge of the topic “How can diet & exercise affect HTN?” with 8 questions. They started with a high level of knowledge pre-program (average score of 6.5) and showed significant improvement to a score of 7.4 at 1 week post-program (*P*=0.0387; 7.4 for n=18; Figure 5A). The topic “What is BP & HTN?” was assessed with 8 questions. The students started with an average score of 3.0 pre-program that rose significantly to a score of 5.3 at 1 week post-program (*P*<0.0001; 5.4 for n=18; Figure 5B). On the 5 questions assessing the topic “How can HTN affect your body?”, students scored an average of 1.9 pre-program and significantly increased their score to 3.5 at 1 week post-program (*P*=0.0062; 3.3 for n=18; Figure 5C).

In the self-efficacy assessment (9 statements), the subsequent cohort improved from a pre-program average score of 4.4 to a score of 5.9 at 1 week post-program, and the difference trended toward significance (*P*=0.0505; 5.9 for n=18; Figure 5D).

The average scores for the 4 statements assessing the students’ attitudes on taking a BP measurement showed no significant difference between the average score of 2.4 pre-program and the score of 2.8 at 1 week post-program (2.9 for n=18; Figure 5E).

#### BP Competency Rubric Data.

Ten students completed the BP competency rubric at the 1-week post-program time point. Results are reported as the percentage of students who correctly demonstrated the step. Of the 8 rubric steps, the subsequent cohort scored >55% on 3 steps: “Choose the right cuff size?” (100% [10 students]), “Put the cuff in the correct orientation?” (100% [10 students]), and “Know the top number is systole?” (60% [6 students]) (Figure 4B).

## DISCUSSION

For this study, elementary school students were exposed to basic HTN knowledge and prevention techniques. The data provide sufficient evidence to conclude that Teach BP was effective at increasing students’ awareness of HTN.

The most significant improvements were seen in students’ understanding of the topics “What is BP & HTN?” and “How can HTN affect your body?”. At the first post-program assessment time point, average scores on “How can HTN affect your body” rose by an average of 92.1% (100% in the original cohort and 84.2% in the subsequent cohort), and “What is BP & HTN?” scores rose by an average of 62.7% (48.8% in the original cohort and 76.7% in the subsequent cohort), suggesting both a lack of pre-program knowledge and the effectiveness of the lessons. These findings align with a similar study from Singapore where students were taught a BP program and demonstrated an increased ability to identify a normal BP.^26^ Findings across both studies suggest that students respond favorably to clear definitions of normal and abnormal BP readings.

Overall, the original cohort retained knowledge at the post-program time points. From the 1-day post-program assessment to the 6-week post-program assessment, the average score for “What is BP & HTN?” decreased by 6.6% (<1 point lower), and the average score for “How can HTN affect your body?” decreased by 26.3% (1 point lower). This result differs from a similar study conducted in South India where middle school students’ knowledge either increased or plateaued at 1 month and 3 months post-program.^25^ To target knowledge retention, the Teach BP team will consider incorporating an annual refresher course in the curriculum.

The average score for “How can diet & exercise affect HTN?” responses increased significantly in the subsequent cohort but not in the original cohort. This finding differs from the comparable survey results from the study in South India, where students demonstrated a significant increase in knowledge related to risk factors.^25^ This contrast may be the result of required diet and exercise curriculum standards set by the National School Lunch Program. The United States has required national nutrition standards for students since 1946, while similar legislation was not implemented in India until 2020.^32,33^ The mandated nutrition and exercise education in the United States may also explain the high baseline scores of both the original cohort and the subsequent cohort in their responses to questions about “How can diet & exercise affect HTN?”. The discrepancy between the 2 studies could also reflect differences in study design. While the South India curriculum centered on areas for improvement following the baseline examination, Teach BP is a fixed curriculum that does not adjust based on students’ knowledge gaps.

The novel aspect of the Teach BP curriculum is breaking down the BP measurement process into a rubric that children can follow. Comparable studies have not reported students’ ability to accurately take BP. The American Heart Association (AHA) BP measurement instructions include telling a patient to relax, choosing the right cuff, placing the cuff properly, and recording the results. In the original cohort, more than 50% of students completed each of these steps. In the subsequent cohort, at least 50% of students completed 3 of these steps (only 30% correctly recorded the results). According to the rubric, students demonstrated knowledge beyond AHA guidelines, including the ability to label and define the numbers on the machine. In both cohorts, only 1 step not outlined by the AHA—“Know that diastole is when the heart is relaxing”—had <50% of the students answer correctly (42.3% in the original cohort and 30% in the subsequent cohort). These findings indicate that in future iterations of the Teach BP curriculum, more emphasis can be placed on ensuring the comprehension of systole and diastole.

Because elevated levels of self-efficacy are associated with greater levels of academic performance and better health outcomes,^34-36^ the Teach BP team investigated students’ self-efficacy regarding BP at the pre-program and post-program time points. The 6-week post-program assessment data showed no significant change in self-efficacy in the original cohort, but the change in attitudes on taking a BP measurement was significant (*P*=0.0180). Following changes to the surveys, students in the subsequent cohort demonstrated an increase in attitudes and a nearly significant increase in self-efficacy (*P*=0.0505). We do not have enough data to make a conclusive assessment of self-efficacy levels. Teach BP will continue to reevaluate methods of measuring self-efficacy to capture the most accurate representation of student confidence when taking a BP.

The success of Teach BP supports that health interventions centered on elementary students can cultivate awareness of HTN, the number one modifiable risk factor for cardiovascular health. Future directions for this program include expanding the scope to include other schools and integrating the program in the greater community. Specifically, the team hopes to encourage students to share BP knowledge with their families and increase awareness of HTN across multiple generations.

### School Health Policy Implications

US schools that receive federal funding for free or reduced-price student lunches are required to implement a wellness policy to promote nutrition education, exercise, and wellness activities.^37^ Almost 100,000 public and private schools receive this funding, with indications that the Healthy, Hunger-Free Kids Act of 2010 improved the nutritional intake for children eating school lunch; however, the foods served in schools focused on reducing childhood obesity.^38^ While obesity is a major public health concern for children, considering other chronic diseases such as HTN is also important. This gap highlights an opportunity to implement additional programs designed to increase student awareness about HTN, a disease that kills almost 700,000 Americans per year.^39^

Teach BP offers evidence that health education practices outlined in the Whole School, Whole Community, Whole Child model can improve student awareness of chronic diseases prevalent in their community.^40^ In 2014, the Centers for Disease Control and Prevention introduced the Whole School, Whole Community, Whole Child model that encourages schools to integrate community-based health interventions that are culturally relevant and responsive to local needs into school settings.^22,40^ The model emphasizes the importance of collaboration between the education sector and the broader community to support student health and academic achievement. While widespread implementation of the Whole School, Whole Community, Whole Child model has been slow, Teach BP offers evidence that embracing community-based health interventions in schools can increase HTN awareness in young people and teach them how to identify and prevent the disease. By adopting aspects of the Whole School, Whole Community, Whole Child model, schools can develop a more comprehensive approach to promoting health and well-being among students.

### Limitations

Several limitations affect the power of this quasi-experimental study. First, the maximum numbers of student participants in phase I and phase II were 30 and 52, respectively, and the numbers of students who completed the surveys and rubrics were not uniform at post-program time points. Student attendance varied, and students were not tracked with identifiers, so Teach BP instructors were unable to identify the students who missed parts of the program instruction. Thus, some students may have experienced more of the curriculum than others. The rubric was conducted one-on-one with students, and because of the fewer number of Teach BP instructors than students, not every student was able to complete the rubric with an instructor during the allotted hour. Second, this study did not have a control group, as the research team did not want to exclude any students from the program. Third, the study was conducted in a faith-based private school with a majority Black and low-income population. As a result, findings may be generalizable only to schools with similar demographics. Additionally, students were assessed using slightly modified methodology, as minor improvements were made to the curriculum and surveys for clarity. Different members of the Teach BP team conducted the BP competency rubric assessment, so some variability in what was considered correct or incorrect is possible. Finally, this study failed to measure if students shared their HTN awareness with their caregivers or with community members outside of the classroom. Moving forward, we plan to repeat this study with additional metrics to observe if students transfer HTN knowledge to their community.

## CONCLUSION

This Teach BP study demonstrated the ability to teach elementary school students about HTN, healthy lifestyles, and BP screening procedures; however, we identified opportunities to improve the teaching of specific BP terminology and to engage student caregivers. Future directions entail expanding this program to other schools and increasing the interaction between Teach BP and families.

## Notes

*This article meets the Accreditation Council for Graduate Medical Education and the American Board of Medical Specialties Maintenance of Certification competencies for Patient Care and Medical Knowledge.*
